# Epidemiological characterization of COVID-19 in displaced populations of South Sudan

**DOI:** 10.11604/pamj.supp.2022.42.1.33767

**Published:** 2022-06-09

**Authors:** Joseph Francis Wamala, Fredrick Loro, Simon John Deng, Kibebu Kinfu Berta, Argata Guracha Guyo, Allan Mpairwe, Fabian Ndenzako, John Pasquale Rumunu

**Affiliations:** 1 Emergency Preparedness and Response, World Health Organization Country Office, Juba, South Sudan,; 2 World Health Organization Regional Office for Africa, Nairobi Hub, Kenya,; 3 Ministry of Health, Juba, South Sudan

**Keywords:** Epidemiology, COVID-19, displaced populations

## Abstract

**Introduction:**

South Sudan is facing a protracted humanitarian crisis with increasing population vulnerability. The study aimed to describe the epidemiology of COVID-19 in displaced populations in South Sudan.

**Methods:**

the study involved the internally displaced populations (IDP) in Bentiu IDP camp, South Sudan. This was a descriptive cross-sectional study involving individuals that met the COVID-19 probable and confirmed case definitions from May 2020 to November 2021. Case data were managed using Microsoft Excel databases.

**Results:**

the initial COVID-19 case in Bentiu IDP camp was reported on 2 May 2020. The overall cumulative attack rate (cases per million) was 3,230 for Bentiu IDP and 1,038 at the national level. The COVID-19 Case Fatality Ratio (CFR) among the IDPs was 19.08% among confirmed and 1.06% at the national level. There was one wave of COVID-19 transmission in the IDPs that coincided with the second COVID-19 wave in South Sudan for the period May 2020 to November 2021. Adult males aged 20-49 years were the most affected and constituted 47.1% of COVID-19 cases. Most severe cases were reported among adults 60-69 years (53%) and ≥ 70 years (80%). The risk of COVID-19 death (deaths per 10,000) increased with age and was highest in patients aged ≥ 60 years at 64.1. The commonest underlying illnesses among COVID-19 deaths was HIV-related illness, heart disease, and tuberculosis.

**Conclusion:**

COVID-19 constitutes a significant impact on internally displaced populations of South Sudan. The COVID-19 response in displaced populations and the high-risk groups therein should be optimized.

## Introduction

South Sudan has braced a protracted humanitarian crisis characterized by cycles of violence, displacement, severe food insecurity, flooding, and disease outbreaks like cholera, yellow fever, hepatitis E virus, and other public health emergencies [1-4]. These persistent shocks have had a compounding effect, eroded coping capacities, increased vulnerabilities, and increased the risk of excess morbidity and mortality [1]. The internally displaced population (IDP) in South Sudan has been increasing and is estimated at 2 million people [1]. Bentiu IDP camp was established in 2014 as a protection of civilians camp but transitioned into an IDP camp in March 2021 and is the largest IDP camp in the country with an estimated population of 107,130 people [5]. In 2021, an outbreak of hepatitis E virus was confirmed in Bentiu IDP camp and this was attributed to inadequate access to safe water and sanitation that was below sphere standards [6]. While a multi-cluster hepatitis E virus outbreak response is underway these efforts have been constrained by new displacements into the camp due to the devastating flooding that has affected over 200,000 people in Unity state [7].

It is within this context that South Sudan is responding to the raging COVID-19 pandemic that has not spared displaced populations in South Sudan. South Sudan was one of the last countries to confirm Coronavirus disease 2019 (COVID-19) and reported its first case on 5th April 2020. As of 10th December 2021, a total of 12,873 confirmed COVID-19 cases including 133 deaths (Case Fatality Ratio (CFR) 1.03%) have been reported [8]. COVID-19 patients manifest with respiratory illness that varies from asymptomatic or mild illness to moderate and severe disease requiring hospitalization [9,10].

The impact of COVID-19 was therefore expected to be unprecedented in low-income settings and displaced populations [11]. The adverse COVID-19 outcomes would be driven by high transmission in extended households, overcrowding in IDP camps, inadequate access to water and sanitation, and super spreading events within the context [12]. Moreover, the high burden of noncommunicable diseases like uncontrolled hypertension, malnutrition, tuberculosis, and HIV in low income and forced displacement settings was expected to increase the risk of severe disease, demand on admission and critical care, with the potential of overburdening systems and constraining access to routine healthcare and immunization for common causes of morbidity and mortality in children and women of childbearing age [13].

The overall control of COVID-19 is premised on strong surveillance and testing capacities, isolation and effective treatment of cases, quarantine of contacts, vaccination with priority to high risk groups, and communitywide implementation of public health social measures like stay home lockdowns, barring of congregations and superspreading events, mask use in closed and public places, regular hand washing, and social distancing [14]. However, in low income, fragile, and vulnerable settings, extended population wide COVID-19 restrictions have been associated with negative social and economic implications with erosion of livelihoods and coping capacities [15]. Hence the alternative of time limited movement restrictions, community led shielding of high risk individuals, self-isolation of mild to moderately ill individuals, and physical distancing were proposed as balanced approaches in low income, fragile, and vulnerable settings [15]. More importantly, the equitable distribution of countermeasures like vaccines, diagnostics, and therapeutics is critical for optimal COVID-19 control in forced displacement settings [15].

We present here the epidemiology of COVID-19 in displaced populations in South Sudan as a precursor for evidence based and targeted pandemic response. The information generated will facilitate equity in the distribution of medical and public health countermeasures to displaced populations and the high-risk groups therein to avert catastrophic outcomes.

## Methods

**Study area and population:** the study was conducted in South Sudan and involved the internally displaced population living in Bentiu IDP camp, Rubkona county, Unity State. The camp was established in December 2013 and as of June 2021, the registered population stood at 107,130 individuals living in 15,716 households [5]. As part of the COVID-19 pandemic response, suspect COVID-19 cases were identified, tested, isolated, and followed up guided by the national standard operating procedures (SoP) for COVID-19 surveillance [16]. The present study identified all the confirmed COVID-19 cases reported by the Ministry of Health (MoH) in Bentiu IDP camp from 5th April to 7th November 2021. The South Sudan COVID-19 surveillance (SOP) defined COVID-19 cases as follows [16]. A suspect COVID-19 case was defined as any person presenting with at least two of the following symptoms: fever (>38°C), chills, rigors, myalgia, headache, sore throat, fatigue, vomiting, diarrhea, sudden loss of taste, sudden loss of smell; OR at least one of the following symptoms: severe cough, shortness of breath, or difficulty breathing; OR severe respiratory illness; AND no alternative more likely diagnosis.

A probable COVID-19 case was defined as a suspect case for whom testing for COVID-19 was inconclusive OR a suspect case for whom testing could not be performed for any reason. A confirmed COVID-19 case was defined as a person with laboratory confirmation of SARS-CoV-2 (by Nucleic Acid Amplification Test (NAAT), irrespective of clinical signs and symptoms OR a person with a positive SARS-CoV-2 Antigen-RDT AND meeting either the probable or suspect case definition OR an asymptomatic person with a positive SARS-CoV-2 Antigen-RDT who is a contact of a probable or confirmed case. A COVID-19 death was defined as a death from a clinically compatible illness in a probable or confirmed COVID-19 case, unless there is a clear alternative cause of death that cannot be related to COVID-19 for example trauma. There should be no period of complete recovery between illness and death [17,18].

**Inclusion criteria:** all individuals who met the case definition of a COVID-19 probable or confirmed case and a COVID-19 death in the Bentiu IDP COVID-19 case and mortality database from 5th April 2020 to 7th November 2021 were included in the study.

**Exclusion criteria:** all individuals who did not meet the case definition of a COVID-19 probable or confirmed case definition and a COVID-19 death that were not listed in the Bentiu IDP COVID-19 case and mortality database from 5th April 2020 to 7th November 2021 were excluded from the study.

**Study design:** this was a descriptive cross-sectional study involving all individuals that met the inclusion criteria. The study used both quantitative data from the national and Bentiu IDP COVID-19 case and mortality database and other descriptive and context information from the national weekly COVID-19 epidemiological bulletin and other state level COVID-19 and humanitarian situation updates.

**Sample size and methods:** all the individuals that met the COVID-19 probable and confirmed case definition and who were included in the Bentiu IDP COVID-19 case and mortality database during the study period were included in the study.

**Data collection:** all case investigations and data collection were integrated into and started with the investigation of the respective suspect and probable COVID-19 cases using the national COVID-19 case investigation form that also documented clinical outcome as recovered or died. Communities reported suspect COVID-19 cases through a telephone hotline, to the nearest health facility, or to designated COVID-19 sentinel sites, or other designated COVID-19 testing sites including private health facilities and laboratories. Probable or confirmed COVID-19 were also identified among contacts to confirmed or probable COVID-19 cases, as part of pretravel screening, or though screening in high-risk populations like workplaces (health facilities), schools, other institutions, and COVID-19 field investigations by rapid response teams. COVID-19 case data was entered into paper-based COVID-19 case investigation form. Verbal autopsies were conducted for probable COVID-19 deaths. Electronic entry of case based data into a Microsoft Excel based database occurred at designated COVID-19 designated GeneXpert sites and at the Data Management Unit (DMU) in the Public Health Emergency Operations Center (PHEOC) in Juba. As part of the current study, the COVID-19 weekly epidemiological bulletins and humanitarian situation reports were reviewed to obtain descriptive and contextual information requited to explain the observed epidemiological statistics.

**Data management and analysis:** all COVID-19 case, and mortality data were managed using Microsoft Excel databases at the national and State level. However, at the time of writing this paper, the COVID-19 DHIS 2 module was being rolled out to support all the COVID-19 pandemic health information needs including the management of COVID-19 case based data. We used the Bentiu IDP, and national level COVID-19 Microsoft excel database files to summarize the frequency distribution of cases and deaths by gender, age, and time. To assess the severity of illness by age, we ran the frequency distribution of case admissions to Bentiu IDP isolation facility by 10-year age groups. The risk of death from COVID-19 by age was computed as the number of deaths per 10,000 Bentiu IDP population for each of the 10-year age categories. We used the Bentiu IDP mortality line list to compute the overall crude mortality rate (CMR) (deaths per 10,000 population per day) and the under-five mortality rate (U5MR) (deaths per 10,000 population of under-fives per day) in the IDP camp [19]. To assess the impact of COVID-19, we compared the CMR and U5MR prior to the pandemic (in 2019) to the rates during the pandemic (2020 and 2021). We ran a frequency distribution to identify the common underlying illnesses among deceased COVID-19 cases in the IDP camp.

**Quality control:** trained COVID-19 response teams including rapid response teams, contact tracing teams, sentinel site teams, and clinicians at designated public and private clinics and laboratories collected case based data using a simplified COVID-19 case investigation form. A dedicated team of Ministry of Health data managers in the PHEOC Data Management Unit were mandated to review and update the records for completeness and to clean the data on a daily basis. The case based records were reviewed for completeness and consistence clinical and epidemiological information with the COVID-19 case and death classification.

**Ethics approval and consent to participate:** the study used existing COVID-19 case based data and context information from routine COVID-19 epidemiological reports and humanitarian situation reports. The study is therefore regarded as operational research for which administrative clearance was provided by the national COVID-19 incident manager to foster evidence based response to the pandemic. The paper was also cleared by WHO under ePub number (ePub-IP-00332814-EC). Moreover, the Research Ethics Review Board of the Ministry of Health provided clearance for the publication of manuscript under (MoH/RERB/D.03/2022) clearance number.

## Results

The initial COVID-19 case in Bentiu IDP camp was reported on 2th May 2020 with a total of 346 cases reported up to 7th November 2021. The COVID-19 cases reported in the IDP camp included 309 confirmed cases, 37 probable cases, and 66 deaths. The COVID-19 Case Fatality Ratio (CFR) among the IDPs was 19.08% among confirmed and probable cases and 9.39% among confirmed cases. During the corresponding period, 12,514 confirmed cases including 133 deaths were reported at the national level with a CFR of 1.06%. Cumulative attack rate (cases per million) was 3,230 for Bentiu IDP and 1,038 at the national level Table 1.

**Table 1:** COVID-19 confirmed cases by location, 5 April 2020 to 7 November 2021

Location	COVID-19 status	Alive	Died	Total cases	Cumulative attack rate (cases/ million)	Case Fatality Ratio (CFR)
Bentiu IDP	Confirmed	280	29	309	2,884	9.39%
	probable	0	37	37	345	100.00%
	Probable/confirmed	280	66	346	3,230	19.08%
National level	Confirmed	12,381	133	12,514	1,038	1.06%

**Figure 1:**
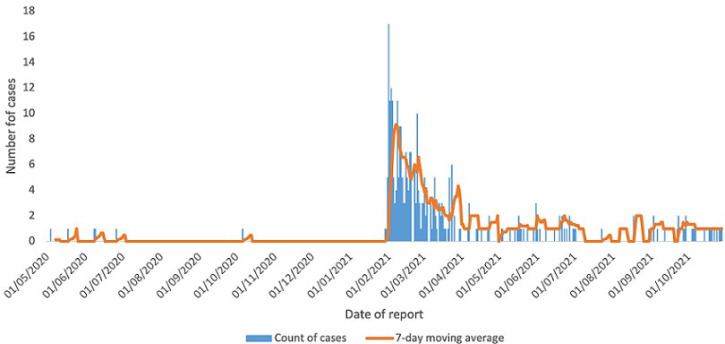
COVID-19 confirmed cases in Bentiu IDP, week 18, 2020 to week 43, 2021

**COVID-19 case trends:** as seen from Figure 1, the initial confirmed COVID-19 cases were reported in week 18, 2020, four weeks after the initial case was notified in South Sudan. The initial case reported history of travel outside the IDP camp within 14 days of illness onset. Sporadic transmission continued up to week 4, 2021 with no obvious travel history and minimal secondary cases among investigated contacts. Steady and clustered transmission started in week 5, 2021, with the 7-day moving average rising steeply from 1 case at the beginning of week 5, 2021 to a peak of 9.1 cases at the end of week 6, 2021. The COVID-19 cases in the IDP camp declined steadily thereafter, the decline coinciding with intensification of COVID-19 control measures that included an imposition of a time limited partial lockdown with restrictions on travel, gatherings, closure of schools and churches, as well imposition of curfew hours.

**Figure 2:**
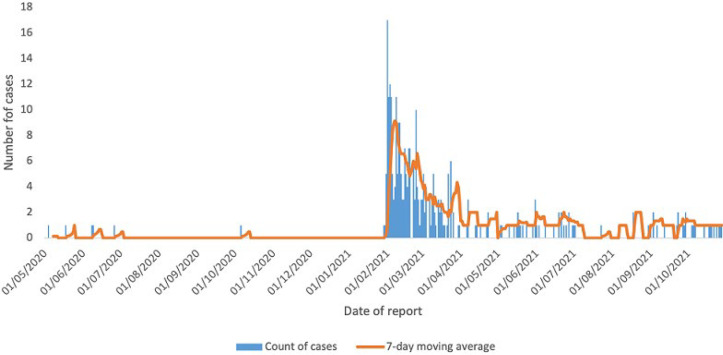
COVID-19 confirmed cases in South Sudan, week 1, 2020 to week 43, 2021

At the national level, the initial cases were reported in week 4 of 2020 and were associated with travel outside the country (Figure 2). Sporadic transmission with no clustering and few secondary cases among investigated contacts continued up to week 18 of 2020 when cases started rising steadily (Figure 2). The cases then rose from 23 cases per week in week 18, 2020 to a peak of 447 cases in week 22 of 2020, also the peak of the first wave of transmission in the country (Figure 2). The second wave of transmission in South Sudan started in week 3, 2021 reaching a peak of 1,369 cases in week 7, 2021 with the cases declining steadily thereafter (Figure 2). The peak of the second wave of transmission in South Sudan exceeded the first wave by 206% and occurred one week after the peak transmission in Bentiu IDP. The second wave of COVID-19 transmission was associated with a steep rise in cases and also coincided with the isolation of the eta SARS-CoV-2 variant in South Sudan.

**Figure 3:**
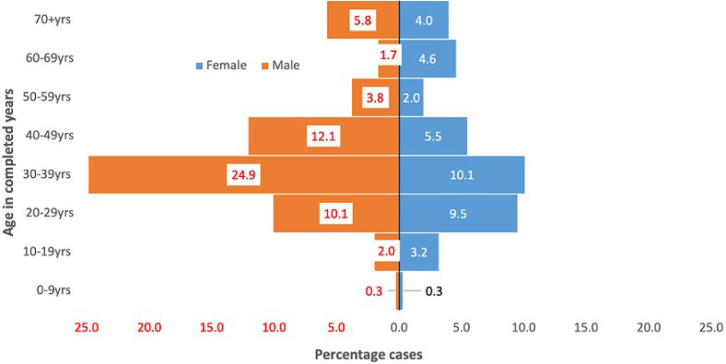
COVID-19 cases in Bentiu IDP camp, Unity state, 2 May 2020 to 7 November 2021

**Case distribution by sex and age:** most of the COVID-19 cases in Bentiu IDP were reported among males 210 (60.7%). During the same period, 6,919 (71.5%) of the cases were reported among the males countrywide. Most of the COVID-19 cases in Bentiu IDP camp were reported among males aged 20-49 years who accounted for 47.1% of the total cases. Females 20-49 years accounted for 25.1% of the cases reported in Bentiu IDP camp (Figure 3). Most of the cases reported at the national level were males 20-49 years who constituted 56.1% of the cases reported countrywide. Females 20-49 years accounted for 21.7% of the cases reported countrywide.

**Severity of COVID-19 illness:** admissions to Bentiu IDP isolation facility were limited to moderately ill patients with underlying chronic illness as well as severely ill and critically sick patients. Of the 309 patients with documented admission status, 104 (34%) were admitted to the isolation facility (Table 2). The age groups with the highest proportion of cases admitted were 60-69 years 9 (53%) and ≥70 years 16 (80%) (Table 2).

**Table 2:** COVID-19 admission to Bentiu IDP isolation facility, week 18, 2020 to week 43, 2021

Age (yrs)	Admission to hospital		Total cases	Admissions (%)
	No	Yes		
0-9yrs	1	1	2	50%
10-19yrs	9	4	13	31%
20-29yrs	43	21	64	33%
30-39yrs	89	31	120	26%
40-49yrs	37	19	56	34%
50-59yrs	14	3	17	18%
60-69yrs	8	9	17	53%
70+yrs	4	16	20	80%
**Grand Total**	**205**	**104**	**309**	34%

**Figure 4:**
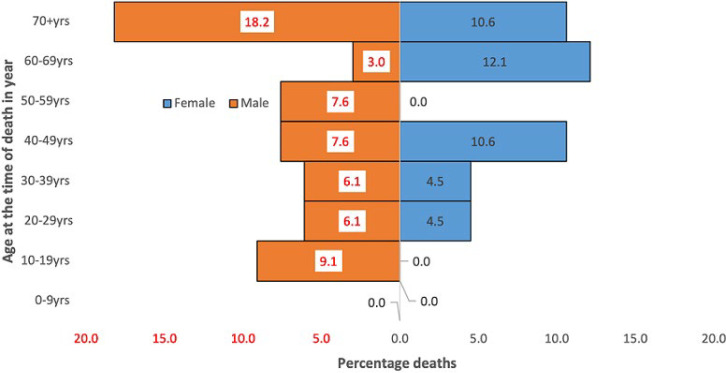
COVID-19 deaths in Bentiu IDP camp, Unity state, 2 May 2020 to 7 November 2021

**Figure 5:**
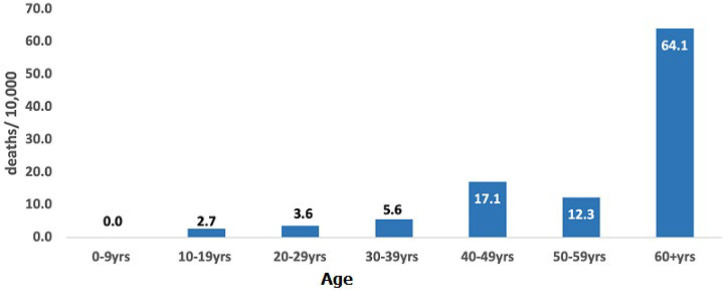
COVID-19 death risk per 10,000 by age, Bentiu IDP, 2 May 2020 - 7 November 2021

**Deaths among COVID-19 cases:** a total of 66 deaths were reported in Bentiu IDP during the reporting period. Most deaths in Bentiu IDP were reported among the males 38 (57.6%). The age group with the highest proportion of COVID-19 deaths was ≥60 years with 29 (78.4%). Males aged 70 years and above accounted for the highest proportion of deaths 19 (18.2%), followed by females aged 60-69 years of age 8 (12.1%) (Figure 4). The risk of COVID-19 death (deaths per 10,000) rose with age from 2.7 among the 10-19 year old patients to 64.1 in patients aged ≥ 60 years (Figure 5). Among the admitted COVID-19 cases, the highest proportion of deaths were reported among the 50-59 year age group (66.7%) and 60-69 year age group (55.6%).

**Crude Mortality Rate (CMR) and Under Five Mortality Rate (UFMR) in Bentiu IDP:** the CMR and U5MR (deaths per 10,000 population per day) were computed each of the years 2019, 2020, and 2021 (up to 7 November 2021). As seen from Table 3, there were marginal increases with no significant changes in the CMR and U5MR in Bentiu IDP during 2019, 2020, and up to 7th November 2021.

**Table 3:** crude and under five mortality rates in Bentiu IDP for 2019, 2020, and Nov 7 2021

Year	<5 yrs	≥5 years	Total deaths	Mid-year population	Number of weeks	U5MR/10,000/day	CMR/10,000 /day
**2019**	197	347	544	100,441	52	0.27	0.15
2020	196	321	517	114,330	52	0.24	0.12
2021	201	385	586	107,130	47	0.29	0.17
**Total deaths**	**594**	**1054**	**1648**				

**Underlying illnesses among COVID-19 deaths:** among the 66 COVID-19 deaths reported in Bentiu IDP during the reporting period, 38 (57.6%) had a documented underlying illness. The most common underlying illnesses reported among COVID-19 deaths in Bentiu IDP included HIV related illness (HRI), heart disease, tuberculosis, renal disease, and stroke (Table 4).

**Table 4:** underlying illnesses in COVID-19 deaths in Bentiu IDP camp, 2 May 2020 - 7 November 2021

S no.	Underlying illnesses	Total deaths	Proportion of deaths
1	Missing	28	42.4%
2	HIV related illness (HRI)	17	25.8%
3	Heart disease	5	7.6%
4	Tuberculosis	3	4.5%
5	Renal disease	3	4.5%
6	Stroke	2	3.0%
7	Anemia	1	1.5%
8	Asthma	1	1.5%
9	Diabetes	1	1.5%
10	Encephalopathy	1	1.5%
11	HBV	1	1.5%
12	Multiple organ failure	1	1.5%
13	Neurosyphilis	1	1.5%
14	Septicemia	1	1.5%
15	Total deaths	66	100.0%

## Discussion

The impact of COVID-19 has been unprecedented with even the best health systems struggling to cope with the demand to optimize surveillance through case finding and investigations, contact tracing, testing, and medical care severe and critical care thus justifying the need for population wide restrictions on travel and gatherings [20]. The World Health Organization has proposed critical response actions for a comprehensive whole of society COVID-19 response [14]. In resource constrained settings, where population wide restrictions are likely to have a huge impact on livelihoods, an approach that entails focus on engaging communities to shield the most vulnerable, self-isolation for mild to moderately ill, and moderate distancing in the community has been proposed [15].

In South Sudan, COVID-19 cases were reported in a population that had already braced multiple shocks of a protracted humanitarian crisis with severe food insecurity, disease outbreaks, floods, and displacement rendering the population vulnerable and at risk of devastating COVID-19 outcomes [1]. In this present study, at least 349 COVID-19 cases were reported in Bentiu IDPs. This translates into a cumulative attack rate (cases per million) of 3,230 for Bentiu IDP. This is three times higher than the national attack rate of 1,038 but many times lower than 5,577, the average cumulative attack rate for the African region that varied from 282 in Niger to 228,794 in Seychelles [21]. The COVID-19 CFR in Bentiu IDP was 19.08%, which much higher compared to the national average of 1.06%, the average for the African region [2.^5^%], and 4.3% in Ethiopia but lower than 59.4% in South Africa [21]. These differences in CFR highlight the elevated vulnerabilities and less than optimal access to COVID-19 interventions including surveillance, testing, case management, and public health social measures. However, the elevated CFR could be explained by low case detection due to low testing rates in South Sudan that stand at 2.3 tests per 10,000 per week and hence leading to undetected cases at community level [8,22].

In terms of COVID-19 trends, COVID-19 cases were not reported in Bentiu IDP until week 18, 2020 and sporadic transmission continued until week 5, 2021 and reaching a peak of 9.1 cases per week in week 6, 2021. This coincided with peak of the second COVID-19 transmission wave in South Sudan and in the African region during which new variants including the eta SARS-CoV-2 variant were detected in South Sudan [23]. The variants detected during the second wave in South Sudan explain in part the more than 200% percent excess transmission registered in comparison to the first wave [23].

Most COVID-19 cases in Bentiu IDP were male (60.7%), a finding that is consistent with gender case distribution at national level and in the African region [8,21]. This is explained in part by better access to testing facilities by men when compared to women, and the better compliance to public health social measures by females when compared to the males. This present study also revealed that young adult males 20-49 years registered most of the cases. A similar distribution was reported at the national level and in the African region with most of these being asymptomatic [8,21]. Hence only 34% of COVID-19 patients in Bentiu IDP were admitted into the isolation facility with most admissions reported in the 60-69 year and ≥70 years age groups who globally, have found to be at a higher risk for severe and critical COVID-19 disease [24]. In the same way, the risk of death from COVID-19 in Bentiu IDP was highest among persons ≥60 years, thus highlighting the need to shield elderly persons in displaced populations [15]. This present study also showed that the most common underlying illnesses reported among COVID-19 deaths in Bentiu IDP were HIV related illness, heart disease, tuberculosis, and stroke. These co-morbidities have been reported to increase the risk of death from COVID-19 and other respiratory illnesses [13,24]. These findings reinforce the need to optimize access to essential healthcare including chronic care in displaced populations to reduce the risk of death from COVID-19.

Given the paucity of the surveillance system in the country and in displaced populations, this present study assessed CMR and U5MR for Bentiu IDP before and during the pandemic. The findings revealed marginal increases with no significant differences in CMR and U5MR in Bentiu IDPs before and during the pandemic. It is therefore unlikely that a significant number of community deaths that could be attributed to COVID-19 occurred and were missed by the surveillance system.

This study had several limitations including the paucity of the COVID-19 surveillance at community and health facility level and the inadequate access to testing in Bentiu IDP camp and the country at large. The stigma associated with COVID-19 also had a negative impact on self-reporting of cases and probable deaths from the communities. These gaps in COVID-19 case and deaths reporting also constrained the identification, and investigation of contacts, as well as investigation and ascertainment of probable COVID-19 deaths in the community. We used the existing integrated disease surveillance and response (IDSR) and Early Warning Alert and Response Network (EWARN) morbidity and mortality data to complement the COVID-19 case and mortality line listing.

## Conclusion

COVID-19 had a significant impact on displaced populations of South Sudan with a cumulative attack rate that that was three times higher than the national average. Similarly, the risk of death among COVID-19 cases among displaced populations was high. The COVID-19 case fatality ratio was 19 times higher in displaced populations when compared to national average. There was only one wave of transmission in the displaced populations. This wave lasted at least four weeks from week 5, 2021 and coincided with the second wave of COVID-19 spread in South Sudan. The exponential transmission during this wave was associated with the isolation of eta as the predominant SARS-CoV-2 variant in the country. Adult males aged 20-49 years were the most affected by COVID-19 illness while adults 60 years and above were found to have a higher risk of severe and critical illness as well as death from COVID-19. The commonest underlying illnesses among COVID-19 deaths in displaced populations included HIV related illness, heart disease, tuberculosis, and stroke. There were marginal increases with no significant differences in crude mortality rate and under five mortality rates in displaced populations before and during the COVID-19 pandemic. There is need to optimize COVID-19 response in displaced populations and the high risk groups therein with special emphasis on improving COVID-19 surveillance, testing, contact tracing, case management, vaccination, improving adherence to the public health social measures, and ensuring access to essential health services to treat other causes of morbidity and mortality.

Availability of data and materials: this study used information and data that is available from the South Sudan Ministry of Health Public Health Emergency Operations Center (PHEOC) and generated in support of the ongoing COVID-19 response in the country.

### What is known about this topic

- The coronavirus disease 2019 (COVID-19) is a respiratory disease caused by Severe Acute Respiratory Syndrome Coronavirus 2 (SARS-CoV-2) virus with a disease spectrum that spans from asymptomatic, mild, and moderate to severe and critical illness. The majority of the patients in the general population present with asymptomatic or mild disease with the risk of adverse COVID-19 outcomes increasing with advancing age and underlying medical illnesses.

### What this study adds

- The study presents the epidemiological characterization of COVID-19 in vulnerable humanitarian populations that are inherently at risk of suffering adverse COVID-19 outcomes. The findings are thus critical for ensuring equity in the distribution of medical countermeasures to avert adverse COVID-19 outcomes.
